# “The Rainbow at the Edge of the Shadow of the Egg”

**DOI:** 10.3201/eid1408.000000

**Published:** 2008-08

**Authors:** Polyxeni Potter

**Affiliations:** * Centers for Disease Control and Prevention, Atlanta, Georgia, USA

**Keywords:** Norman Rockwell, art–science connection, emerging infectious diseases, art and medicine, thermometry, American art, Rockwell and doctors, Rockwell and children, doctor–patient relationship, fine art versus illustration, about the cover

**Figure:**
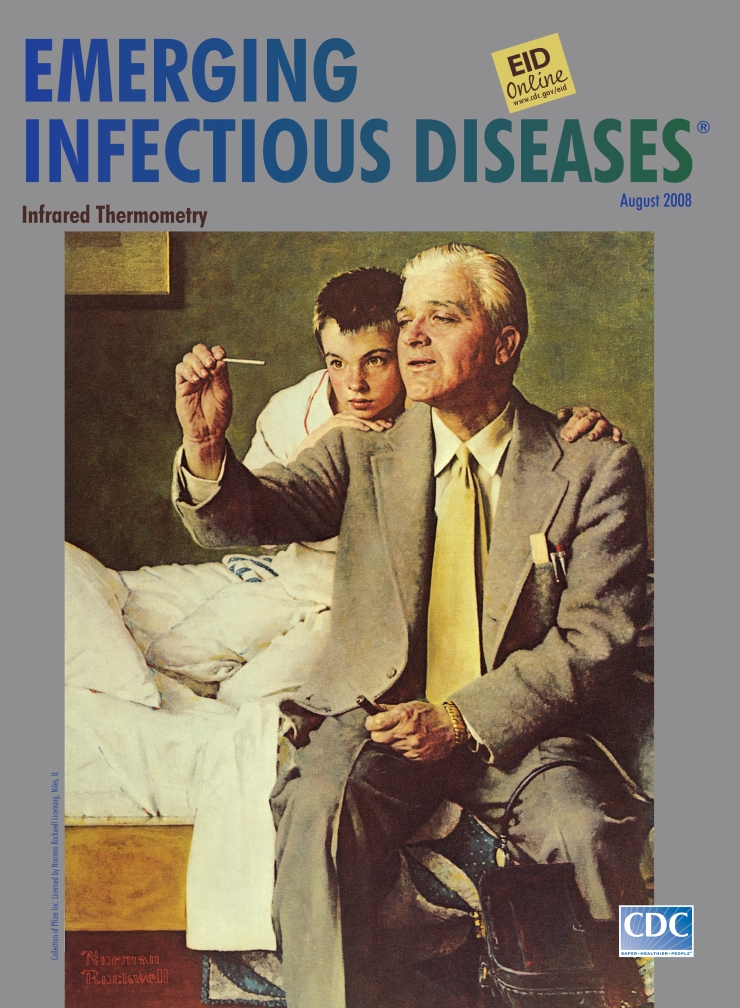
**Norman Rockwell (1894–1978) Doctor and Boy Looking at Thermometer (1954)** Display advertisement for the Upjohn Company. Oil on canvas (101.6 cm × 93.98 cm). Collection of Pfizer Inc. Licensed by Norman Rockwell Licensing, Niles, IL

––John Updike

“Rockwell is terrific. It’s become too tedious to pretend he isn’t,” said art critic Peter Schjeldahl in 1999, summing up recent opinion about this well-loved American artist, whose work was dismissed by the art establishment during his lifetime (*1*). “Without thinking too much about it in specific terms,” Rockwell said of himself, “I was showing the America I knew and observed to others who might not have noticed…. I guess I am a story teller” (*2*).

While hard at work as illustrator for The Saturday Evening Post, Encyclopaedia Britannica, Maxwell House Coffee, Parker Pens, Coca-Cola, Heritage Press, motion picture companies, and the U.S. government, Rockwell was keenly aware of the revolution brewing in the art scene of his day. He thought Picasso was “the greatest,” and in The Connoisseur, painted when he was 68 years old, he showed a middle-aged man clad in a gray suit, fedora and umbrella in hand, pondering a credible representation of a Jackson Pollock canvas.

“I was born on February 3, 1894, in the back bedroom of a shabby brownstone front on 103rd Street and Amsterdam Avenue in New York City,” wrote Rockwell in his autobiography. “I think I’ve always wanted to be an artist…. I drew, then I found I liked to draw, and finally, after I had got to know something about myself and the people and things around me, I found that I didn’t want to do anything else but draw” (*3*).

He studied at the Chase Art School and then went on to the National Academy of Design and the Art Students League, where he worked under Thomas Fogarty, George Bridgman, and Frank Vincent Dumond. “In those days there wasn’t the cleavage between fine arts and illustration that there is today,” Rockwell wrote. “It was the end of what you might call the golden age of illustration.” Frederick Remington had just died, and photographs were crowding the magazine and book publishing scene. Perhaps people had stopped demanding fine illustration, he thought; “You can’t conduct a golden age with a heart of copper” (*3*).

Rockwell was scorned for becoming an illustrator, even by his fellow students. “You know, if I worked as hard as you do, I could be as good as Velásquez,” one of them would joke. “Why don’t you?” Rockwell would respond and return to some vexing problem at hand, how to draw an eye for instance so it did not look “like a fried egg.”

“I can’t say who has influenced me really. Or at least I can’t say *how* the artists I have admired have influenced me…. Ever since I can remember, Rembrandt has been my favorite artist. Vermeer, Breughel, Velásquez, Canaletto; Dürer, Holbein, Ingres as draftsmen; Matisse, Klee―these are a few of the others I admire now. During my student days I studied closely the works of Edwin Austin Abbey, J.C. and Frank Leyendecker, Howard Pyle, Sargent, Whistler” (*3*).

“I had a secret ambition: a cover on the Saturday Evening Post. In those days the cover of the Post was the greatest show window in America for an illustrator…. ‘Must be two million people look at that cover.’” After the Post, offers came from other magazines: Life, Judge, The People’s Popular Monthly. “One of the most difficult problems in painting magazine covers is thinking up ideas which a majority of the readers will understand…. And it’s darned hard to be universal, to find some situation which will strike the farmer, the housewife, the gossip…” (*3*).

During travels to Europe, especially Paris, Rockwell studied modern art and frequented the cafés in Montmartre, absorbing the bohemian scene and its preoccupation with one style over another, now cubism, now color. But it was no use. “I realized that I just didn’t see things as the modern artists did.” Throughout his career, he had periods of self-doubt and inactivity, but he always managed to “rise from the ashes.” As his popularity increased, he became weary of the conventions and restrictions of commercial art so tied to its need to be understood and appreciated by the masses. “I don’t do illustration any more. To tell the truth, I’ve priced myself out of the market. And then, frankly, I’m not too interested. I like to paint my own ideas, tell my own story” (*3*).

He resented being called old-fashioned. “I do ordinary people in everyday situations, and that’s about all I can do…. But, as I say, every so often I get to hankering after immortality and, or so I think at those times anyway, that requires a picture tremendously conceived and tremendously executed.” Along these lines, he painted big ideas: Freedom of Worship, Freedom of Speech, The Problem We All Live With.

“He went beyond the requirements,” argued author, art critic, and fellow chronicler of American life John Updike. “He could have painted with less loving detail. He could have had fewer little anecdotal touches and facial expressions in his work. But he went always to fill the glass to the brim” (*4*). Updike came to understand the power of a detail noticed, when at age 12, he took art lessons. The teacher put an egg in the sun on a piece of white paper and asked him to draw it, then pointed out the detail missed: “the rainbow at the edge of the shadow of the egg” (*5*).

Doctor and Boy Looking at Thermometer, on this month’s cover, shows how Rockwell used detail to capture authenticity. A fold in the skin, the angle of the neck, a crease in the clothes could deliver character. The doctor on the edge of the bed is a prototype. The patient, febrile face animated with curiosity, swollen eye glaring at the thermometer, trusts him. Bemused, the doctor holds up the instrument. The scene is homespun: the quilt, clean sheets, and picture on the wall are balanced by the doctor’s bag and the friendliest suit and tie. In this home theater, the medical emergency is under control.

Modern art was not the only revolution in Rockwell’s time. Much more was changing. The thermometer, in development since antiquity, became a practical tool in 1866, when an instrument that could read body temperature in 5 minutes was available for use. Rockwell’s doctor was likely just as fascinated by it as his young patient. “See,” he was probably saying, “If you hold it just right, you can see the mercury in there.”

The mercury-and-glass thermometer has been replaced by less invasive, more refined versions. Now the race is on for a new, faster instrument to use in the field or hospital for mass screening. Cutaneous infrared thermometry is one such effort (*6*). The elements may all be there, but some fine critical detail is still missing.
